# In Situ Programmable Modulation of Hydrogel Stiffness for Stage‐Adaptive Bone Regeneration

**DOI:** 10.1002/advs.76920

**Published:** 2026-07-31

**Authors:** Yuxin Yang, Fan Yang, Lu Wang, Zongtai Li, Weichang Li, Xinchun Zhang

**Affiliations:** ^1^ Guangdong Provincial Key Laboratory of Stomatology Guanghua School of Stomatology Hospital of Stomatology Sun Yat‐sen University Guangzhou People's Republic of China

**Keywords:** bone regeneration, dynamic stiffness hydrogel, photothermal release, stem cell fate regulation

## Abstract

Bone defect healing is a dynamic process involving changes in the mechanical properties of the extracellular matrix (ECM), which significantly influence cellular behavior and tissue regeneration. In this study, we developed a dynamic stiffness hydrogel system designed to mimic the stiffness variation of the ECM during bone repair. The hydrogel, based on a 3D interpenetrating polymer network, enables in situ modulation of matrix stiffness by adjusting calcium ion concentrations through photothermal effects induced under near‐infrared (NIR) irradiation. The dynamic stiffness of the hydrogel was shown to support stem cell maintenance and promote osteogenic differentiation, aligning with the ECM characteristics observed in natural bone repair processes. Both in vitro and in vivo studies demonstrated that the mechanical cues provided by the hydrogel system significantly impact stem cell stemness and osteogenic potential. Furthermore, the hydrogel exhibited the ability to repair critical‐sized bone defects, underscoring its therapeutic potential. This work introduces a novel platform for bone tissue engineering, combining biomimicry and functional adaptability to optimize bone regeneration and laying the foundation for future clinical applications.

## Graphical abstract

Near‐infrared (NIR) irradiation triggers Ca^2^^+^ release from thermoresponsive nanocarriers, enabling in situ stiffening of an adaptive hydrogel from a soft to a stiff state, thereby sequentially supporting mesenchymal stromal cell stemness and osteogenic differentiation for stage‐adaptive bone regeneration.

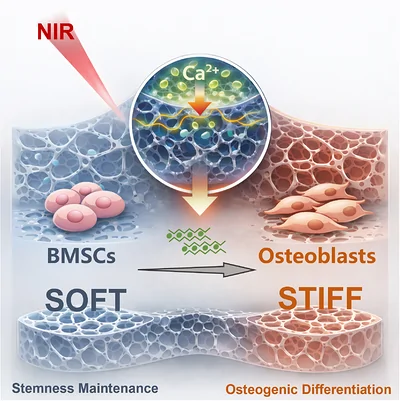

## 1 Introduction

Large bone defects caused by trauma, tumor resection, or congenital disorders remain a major clinical challenge worldwide. More than 20 million patients experience bone loss annually, with approximately five million requiring bone grafting procedures [1]. Although autologous bone grafts remain the clinical gold standard, their use is limited by donor‐site morbidity, infection risk, and insufficient graft availability [2]. Moreover, conventional regenerative strategies rarely consider the dynamic evolution of the extracellular microenvironment during bone healing, which contributes to suboptimal therapeutic outcomes [3]. Therefore, new biomaterial platforms capable of actively matching the time‐dependent requirements of bone repair are urgently needed.

Bone defect repair represents a complex cascade process that involves the precise regulation of biochemical factors, inflammatory responses, and immune cells [4, 5, 6]. Within the regenerative microenvironment, matrix stiffness functions as a key mechanical signal that helps shape the dynamic niche through mechanotransduction. It interacts with biochemical gradients and immune status to collectively direct cell fate and tissue reconstruction [7, 8]. Bone defect healing is a highly dynamic, multistage process initiated by the recruitment of bone marrow–derived mesenchymal stromal cells (BMSCs). During early repair, a compliant microenvironment supports cell proliferation and maintains multilineage potential, whereas progressive matrix stiffening subsequently promotes osteogenic differentiation and tissue maturation [9, 10]. Although BMSCs are regarded as promising seed cells for bone tissue engineering [11], challenges such as cell loss, senescence, and insufficient control over differentiation have limited their translational applications [12, 13, 14, 15]. Increasing evidence from mechanobiology has demonstrated that matrix stiffness is a critical determinant of BMSC fate, influencing cytoskeletal tension, mechanoresponsive signaling, and lineage specification [16, 17, 18, 19, 20, 21, 22]. Soft matrices (0.1–1 kPa) favor neurogenic behavior, intermediate stiffness (8–17 kPa) supports myogenic differentiation, and stiff substrates (>34 kPa) strongly promote osteogenesis [18]. Thus, hydrogels with programmable mechanical properties represent a powerful strategy to guide spatiotemporal cell responses for enhanced bone regeneration.

Hydrogel stiffness is governed by the interplay between covalent crosslinking, coordination bonding, and network topology [23, 24, 25]. Various mechano‐responsive hydrogels have been developed, yet achieving precise in vivo modulation of stiffness after implantation remains a major challenge. Near‐infrared (NIR) irradiation provides several advantages—including deep tissue penetration, spatial precision, and non‐contact remote control—for modulating biomaterial performance in vivo [26, 27, 28, 29, 30]. Photothermal‐responsive hydrogels have been widely explored for controlled drug release and remote actuation [31], while ionic crosslinking systems such as alginate–Ca^2^^+^ networks enable tunable mechanical properties but are generally limited to static or pre‐adjusted stiffness [32]. In addition, thermoresponsive nanocarriers allow temperature‐triggered release of bioactive agents, yet their application in regulating scaffold mechanics remains largely unexplored. Importantly, these functional strategies have mostly been developed independently and rarely address the dynamically evolving mechanical requirements of tissue repair [33]. In this study, we integrate these modules into a unified platform to achieve in situ, programmable, and stage‐adaptive regulation of matrix stiffness in vivo, thereby synchronizing scaffold mechanics with the temporal biological demands of bone healing.

In this study, we propose a dual interpenetrating polymer network (IPN) hydrogel system (PNGMSC) engineered to mimic the dynamic mechanical evolution of the bone repair microenvironment (Scheme 1). The first network is formed through covalent crosslinking of biocompatible methacrylated gelatin (GelMA), acrylamide (AAm), and thermoresponsive poly(N‐isopropylacrylamide) (PNIPAm). Gold nanoparticles (AuNPs), surface‐modified with N,N′‐bis(acryloyl)cystamine (BACA), are incorporated as photothermal triggers to enable NIR‐responsive stiffening. The PNIPAm phase transition synergistically modulates network architecture and enhances calcium‐mediated crosslinking efficiency.

**SCHEME 1:**
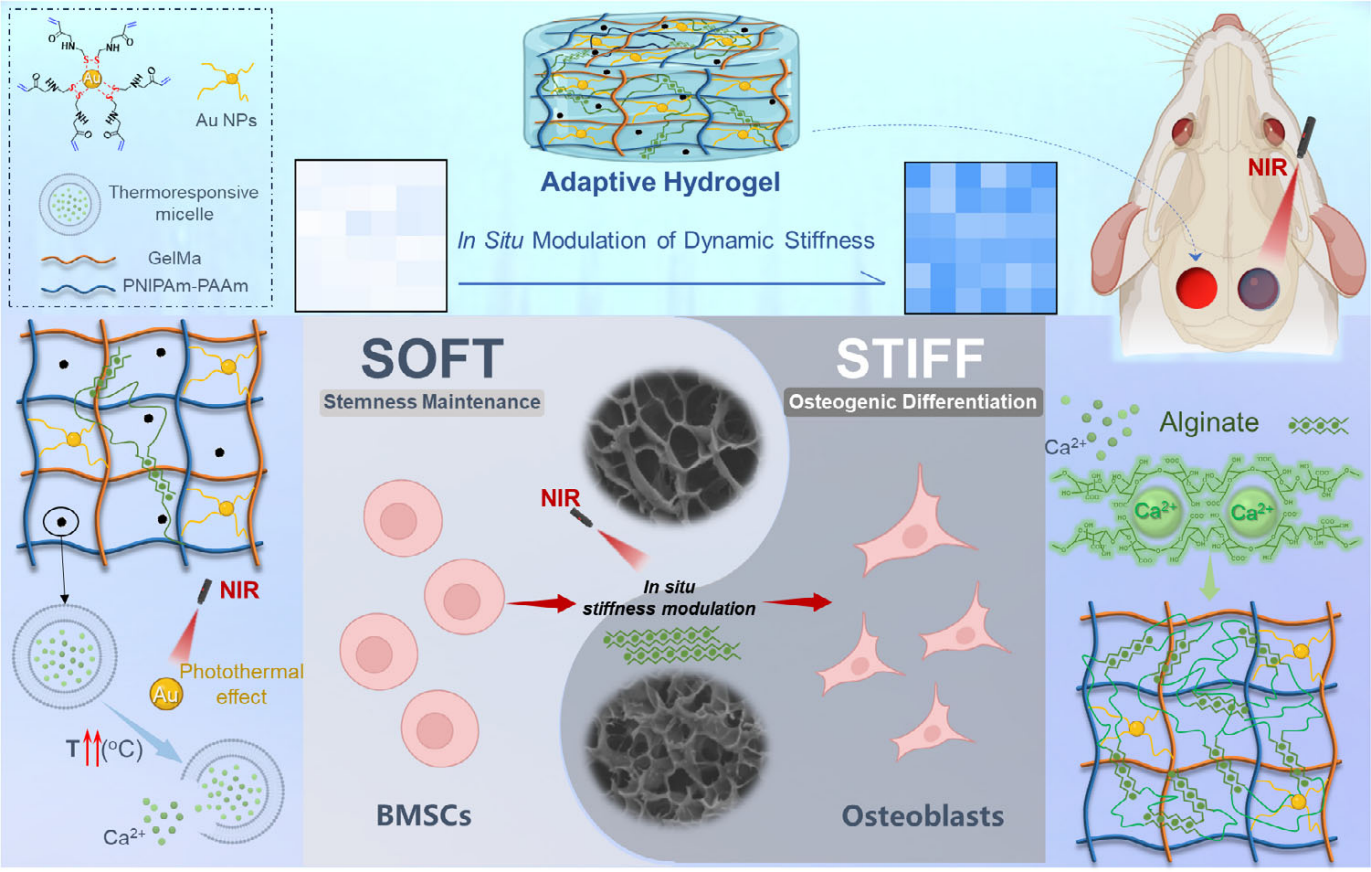
Schematic illustration of the dynamic stiffness–tunable hydrogel system and its role in regulating bone defect repair.

The second network consists of sodium alginate (SA) coordinated with Ca^2^^+^ ions. Modulation of Ca^2^^+^ availability allows fine‐tuning of ionic crosslinking density, thereby adjusting matrix stiffness. To achieve in situ dynamic stiffening, a thermoresponsive nanocarrier loaded with Ca^2^^+^ is embedded within the hydrogel. Upon NIR irradiation, mild local heating induces controlled Ca^2^^+^ release, increasing the crosslinking density of the SA–Ca^2^^+^ network and elevating the overall stiffness of the hydrogel. By adjusting AuNP concentration and irradiation duration, stiffness modulation can be precisely controlled in vivo, enabling stage‐specific regulation of BMSC behavior—supporting proliferation and multilineage potential in early stages and promoting osteogenic differentiation during later stages. Collectively, this adaptive hydrogel platform provides a biomimetic and programmable mechanobiological niche for addressing the clinical challenge of bone defect repair, and offers a promising direction for next‐generation intelligent biomaterials.

## 2 Results and Discussion

### 2.1 Construction and Rationale of a Dynamically Stiffening Hydrogel System

Early‐stage healing after bone injury is characterized by rapid and continuous remodeling of the extracellular matrix (ECM). Immediately following injury, a fibrin‐rich provisional matrix forms and provides initial adhesion sites and mechanical cues for infiltrating cells [34]. As healing progresses, collagen deposition increases cellular traction forces, leading to time‐dependent stiffening of the microenvironment [22, 35, 36, 37, 38]. This evolving biomechanical landscape critically influences BMSC proliferation, maintenance of multilineage potential, and subsequent osteogenic commitment [39, 40, 41, 42]. Therefore, a biomaterial capable of recapitulating the soft‐to‐stiff transition of native ECM is essential for supporting different functional requirements during bone repair.

To determine the mechanical evolution of the defect niche, we established a 5 mm calvarial defect model in SD rats (Figure 1A). The defect region was evaluated on days 1 and 3 post‐injury, corresponding to the formation of fibrin matrix and early granulation tissue, respectively. Atomic force microscopy revealed that the native ECM stiffness increased from ∼7 kPa on day 1 to ∼23 kPa on day 3, which served as the design basis for constructing hydrogels with physiologically relevant stiffness ranges. Based on these requirements, we engineered a 3D interpenetrating polymer network (IPN) hydrogel composed of a covalent primary network and an ionic secondary network (Scheme 1). The primary network consisted of methacrylated gelatin (GelMA), acrylamide (AAm), and thermoresponsive poly(N‐isopropylacrylamide) (PNIPAm). These components were selected due to their excellent biocompatibility, tunable mechanics, and established use in stem‐cell‐related biomaterials. Gold nanoparticles functionalized with N,N′‐bis(acryloyl)cystamine (BACA) (Au@BACA) served as multifunctional elements: (i) covalent crosslinkers providing mechanical stability and (ii) photothermal triggers enabling in situ modulation of temperature for controlled calcium ion release.

**FIGURE 1:**
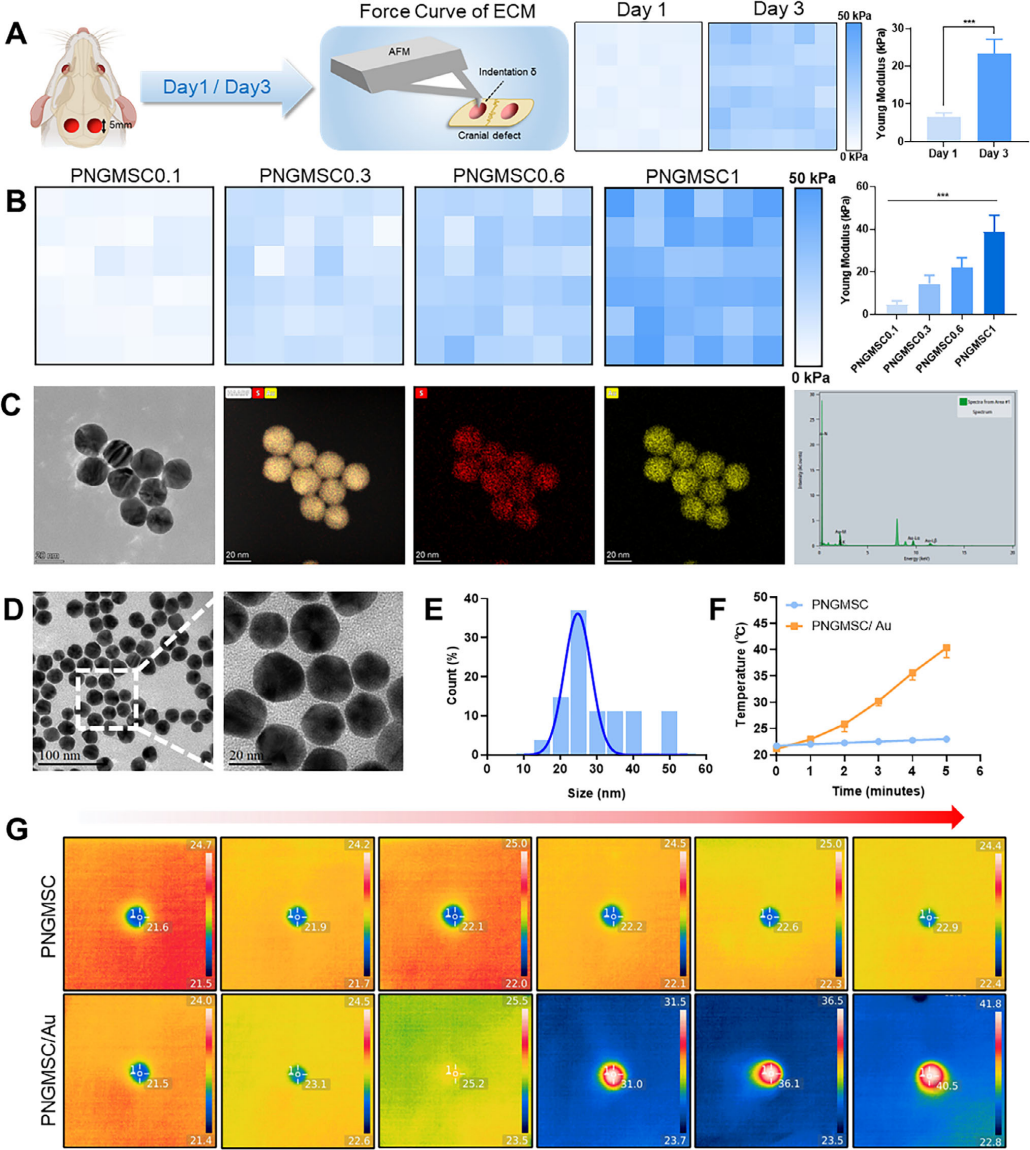
Synthesis and characterization of PNGMSC hydrogels and Au@BACA nanoparticles. (A) Establishment of a rat cranial defect model and AFM‐based measurement of native ECM stiffness at days 1 and 3 post‐injury. Representative force maps and quantitative Young's modulus analysis (n = 36). (B) AFM force mapping and statistical analysis of PNGMSC hydrogels engineered to match early‐stage ECM stiffness (n = 36). (C) Elemental mapping of Au@BACA showing uniform distribution of Au and S elements (scale bar = 20 nm). (D,E) TEM imaging and size distribution analysis of Au nanoparticles (scale bar = 100 nm). (F,G) Photothermal performance of PNGMSC and PNGMSC/Au hydrogels under 808 nm NIR irradiation, with real‐time temperature profiles (n = 3). Data are presented as mean ± SD. Statistical significance: ns, *p* > 0.05; ^*^ *p* < 0.05; ^**^ *p* < 0.01; ^***^ *p* < 0.001.

The secondary network consisted of sodium alginate (SA) coordinated with Ca^2^^+^ to generate a reversible, energy‐dissipative ionic structure. Because ionic crosslink density directly determines network stiffness, varying Ca^2^^+^ concentrations (0.1%, 0.3%, 0.6%, 1%) allowed precise mechanical tuning. Mechanical testing revealed Young's moduli of approximately 4, 14, 22, and 39 kPa, respectively (Figure 1B). Among these, the hydrogels containing 0.1% and 0.6% Ca^2^^+^ closely matched the measured stiffness of native ECM during early bone healing and were therefore defined as PNGMSC‐Soft and PNGMSC‐Stiff for subsequent biological studies.

Gold nanoparticles were selected as the photothermal component due to their excellent biocompatibility, chemical stability, and established biomedical safety profiles [43]. The Au@BACA nanoparticles exhibited uniform spherical morphology and homogeneous surface distribution of sulfur (Figure 1C–E), confirming successful BACA modification. The photothermal performance of the hydrogel was then evaluated under 808‐nm NIR irradiation (2 W/cm^2^). PNGMSC/Au demonstrated a rapid temperature increase, whereas hydrogels without AuNPs showed negligible heating, confirming the essential role of AuNPs in enabling NIR responsiveness (Figure 1F,G). Furthermore, post‐implantation NIR exposure resulted in safe and controllable local temperature elevation (Figure S1A,B), establishing the feasibility of using NIR to trigger on‐demand Ca^2^^+^ release in vivo. To exclude the effect of physiological temperature on non‐responsive Ca^2^^+^ release, constant‐temperature monitoring at 37 °C for 72 h revealed that Ca^2^^+^ release was minimal and remained negligible throughout the testing period (Figure S1C). This system thus integrates a robust covalent skeleton, an ionically tunable dissipative network, and an NIR‐responsive photothermal trigger, providing a platform capable of recapitulating the time‐dependent evolution of ECM stiffness during bone repair and enabling dynamic mechanoregulation of BMSC fate.

### 2.2 Parameter Modulation of the Dynamic Hydrogel System

To realize an in situ stiffness‐adaptive hydrogel capable of matching the evolving biomechanical cues during bone repair, a dual‐network architecture was constructed (Figure 2A (i)). The primary covalent network was formed through copolymerization of AAm, NIPAm, and GelMA, with Au@BACA nanoparticles serving as multifunctional crosslinkers. Concurrently, an alginate (SA)–Ca^2^^+^ ionic network was introduced as a secondary, reversible dissipative network, providing tunable stiffness through modulation of ionic crosslinking density. Thermoresponsive PNIPAm‐b‐PCL micelles preloaded with Ca^2^^+^ were embedded within the hydrogel. Upon near‐infrared (NIR) irradiation, the Au@BACA nanoparticles generated localized photothermal heating, inducing the phase transition of micelles and triggering controlled Ca^2^^+^ release (Figure 2A (ii)). The released Ca^2^^+^ ions strengthened the SA–Ca^2^^+^ coordination network, resulting in a rapid and controllable increase in hydrogel stiffness. Mechanical measurements confirmed that the PNGMSC/Au hydrogel transitioned from approximately 6 to 22 kPa following NIR irradiation (Figure 2A (iii)), enabling a shift from a stemness‐permissive microenvironment to an osteogenic one—consistent with the natural evolution of ECM stiffness during early bone healing.

**FIGURE 2:**
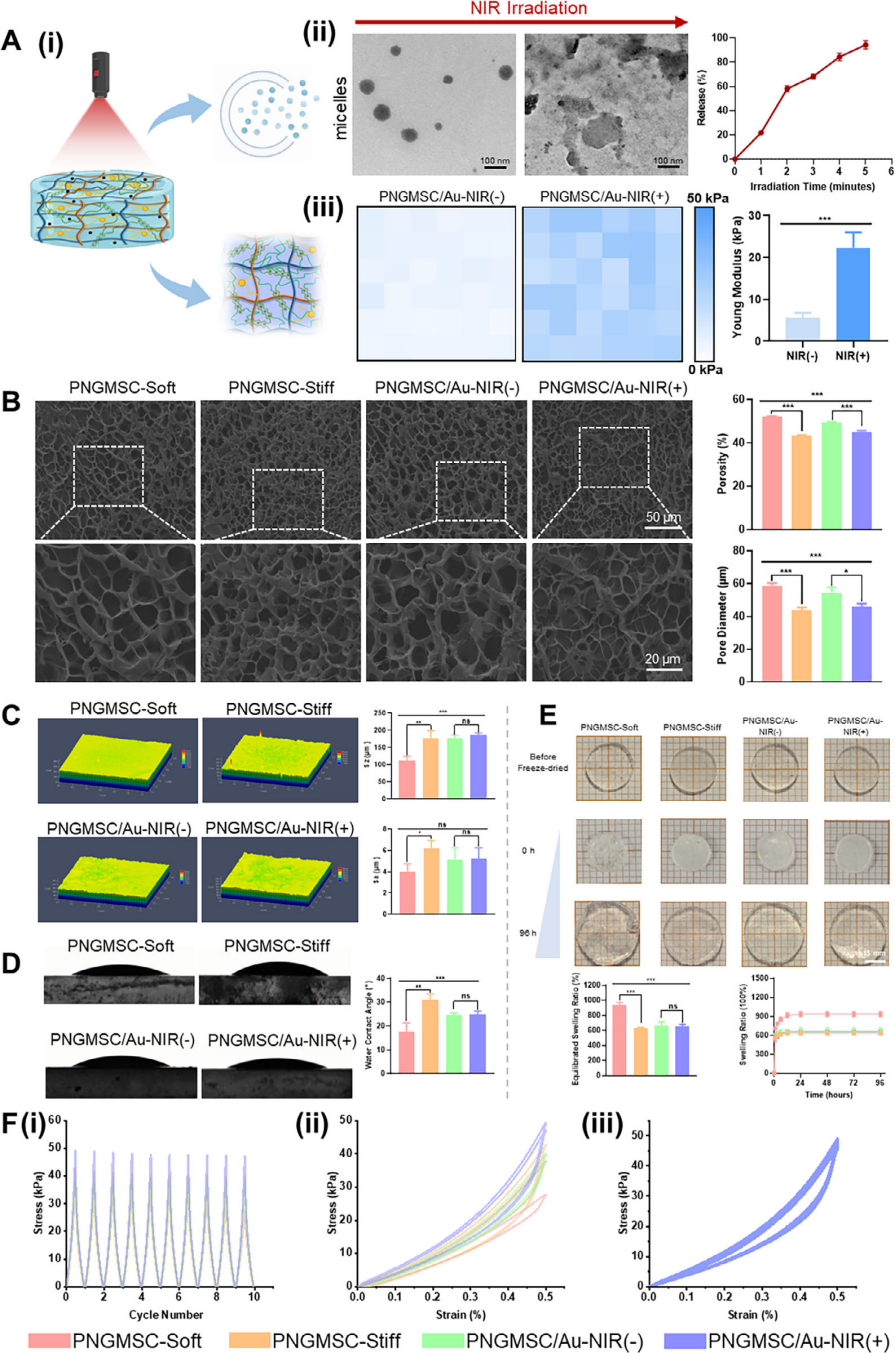
Parameter modulation and physicochemical characteristics of dynamically stiffening PNGMSC hydrogels. (A) (i) Schematic of NIR‐triggered stiffness enhancement through photothermal‐induced Ca^2^^+^ release. (ii) TEM images of calcium‐loaded micelles before/after NIR stimulation and quantitative Ca^2^^+^ release efficiency (n = 3). (iii) AFM force maps and Young's modulus analysis of PNGMSC/Au before and after NIR irradiation (n = 36). (B) SEM images of hydrogel cross‐sections with quantification of pore diameter and porosity (upper scale bar = 50 µm; lower scale bar = 20 µm; n = 3). (C) Surface topography analysis showing Sa and Sz parameters of PNGMSC with varying Ca^2^^+^ contents (n = 3). (D) Water contact angle measurements assessing hydrogel surface hydrophilicity (n = 3). (E) Schematic and quantitative analysis of swelling behavior including equilibrium swelling ratio, swelling kinetics, and swelling mass ratio (n = 4). (F) Mechanical characterization of PNGMSC hydrogels: (i) Compressive stress–strain curves recorded over 10 loading cycles. (ii) Comparison of first and tenth compression cycles. (iii) Fatigue resistance of PNGMSC/Au‐NIR (+) over 50 cycles. Data are presented as mean ± SD. Statistical significance: ns, *p* > 0.05; *p < 0.05; **p < 0.01; ***p < 0.001.

The microstructural differences among hydrogels with varying stiffnesses are illustrated in Figure 2B. Hydrogels with higher Ca^2^^+^ concentrations displayed more compact and denser pore networks, reflecting increased ionic crosslinking. Such microstructural densification contributes to enhanced mechanical stability and improved load distribution within the network. Surface topology analysis further revealed that increasing Ca^2^^+^ levels elevated the surface roughness (Figure 2C), likely due to the formation of Ca^2^^+^‐rich domains within the SA network. This rougher surface is expected to favor protein adsorption and subsequent cell adhesion, thereby supporting early‐stage bone tissue integration. Hydrophilicity assessment showed that the water contact angle increased moderately with increasing Ca^2^^+^ concentration, from 18° at 0.1% Ca^2^^+^ to 31° at 0.6% Ca^2^^+^ (Figure 2D). At low crosslinking density, abundant exposed –COO^−^ groups facilitated strong hydrogen bonding with water molecules, maintaining high surface hydrophilicity. As Ca^2^^+^ crosslinking increased, fewer free carboxyl groups remained available, resulting in a slight decrease in hydrophilicity.

Swelling experiments confirmed that all hydrogels exhibited rapid water absorption due to their intrinsically hydrophilic polymer networks (Figure 2E). Hydrogels with lower Ca^2^^+^ levels displayed higher swelling ratios, attributed to the presence of larger pores and a more loosely packed 3D network. In contrast, highly crosslinked hydrogels formed a dense, compact network with reduced pore size and increased mechanical resistance (Figure 2F). The hydrogel's mechanical properties can be precisely tuned by adjusting Ca^2^^+^ content or by NIR‐triggered Ca^2^^+^ release. Importantly, the baseline stiffness remained stable before NIR illumination, indicating that spontaneous Ca^2^^+^ leakage at physiological temperature is negligible. The designed system thus offers programmable soft‐to‐stiff transitions, enabling phase‐specific regulation of cellular behavior during bone regeneration.

### 2.3 Biocompatibility

The biocompatibility of the hydrogel system was systematically assessed through live/dead staining, CCK‐8 assays, cytoskeletal morphology evaluation, and hemolysis testing. As shown in Figure 3A, BM‐MSCs cultured on all hydrogel formulations maintained high viability and proliferative activity over time, with no statistically significant differences among groups. The introduction of Au@BACA nanoparticles did not induce detectable cytotoxicity (Figure 3D), reflecting the intrinsic biocompatibility of the polymeric components, all of which are widely used in biomedical applications. BM‐MSC morphology was further examined to evaluate early cellular responses to matrix stiffness. Cytoskeletal staining revealed clear stiffness‐dependent spreading behaviors (Figure 3B). Quantitative analysis confirmed that cells on softer substrates (PNGMSC‐Soft) exhibited smaller spreading areas and round, less polarized morphologies, consistent with reduced cytoskeletal tension and limited focal adhesion maturation (Figure 3C). In contrast, cells on stiffer substrates (PNGMSC‐Stiff) displayed significantly larger spreading areas and elongated spindle‐shaped morphologies, indicative of enhanced F‐actin bundling and increased traction forces. These trends are in agreement with classical mechanotransduction principles, whereby substrate stiffness dictates cytoskeletal organization and adhesion structure formation, ultimately shaping cellular behavior. Hemocompatibility is essential for any hydrogel intended for implantation. Hemolysis assays demonstrated that the supernatants of both PNGMSC and PNGMSC/Au hydrogels exhibited absorbance levels comparable to the PBS negative control, with hemolysis rates below 1% in all cases (Figure 3D). These results indicate minimal erythrocyte membrane disruption and confirm excellent blood compatibility of the hydrogel system. Collectively, the dynamic‐stiffness hydrogel system possesses outstanding cytocompatibility and hemocompatibility. The benign cellular responses and negligible hemolytic activity establish a reliable safety profile for subsequent in vivo bone repair applications.

**FIGURE 3:**
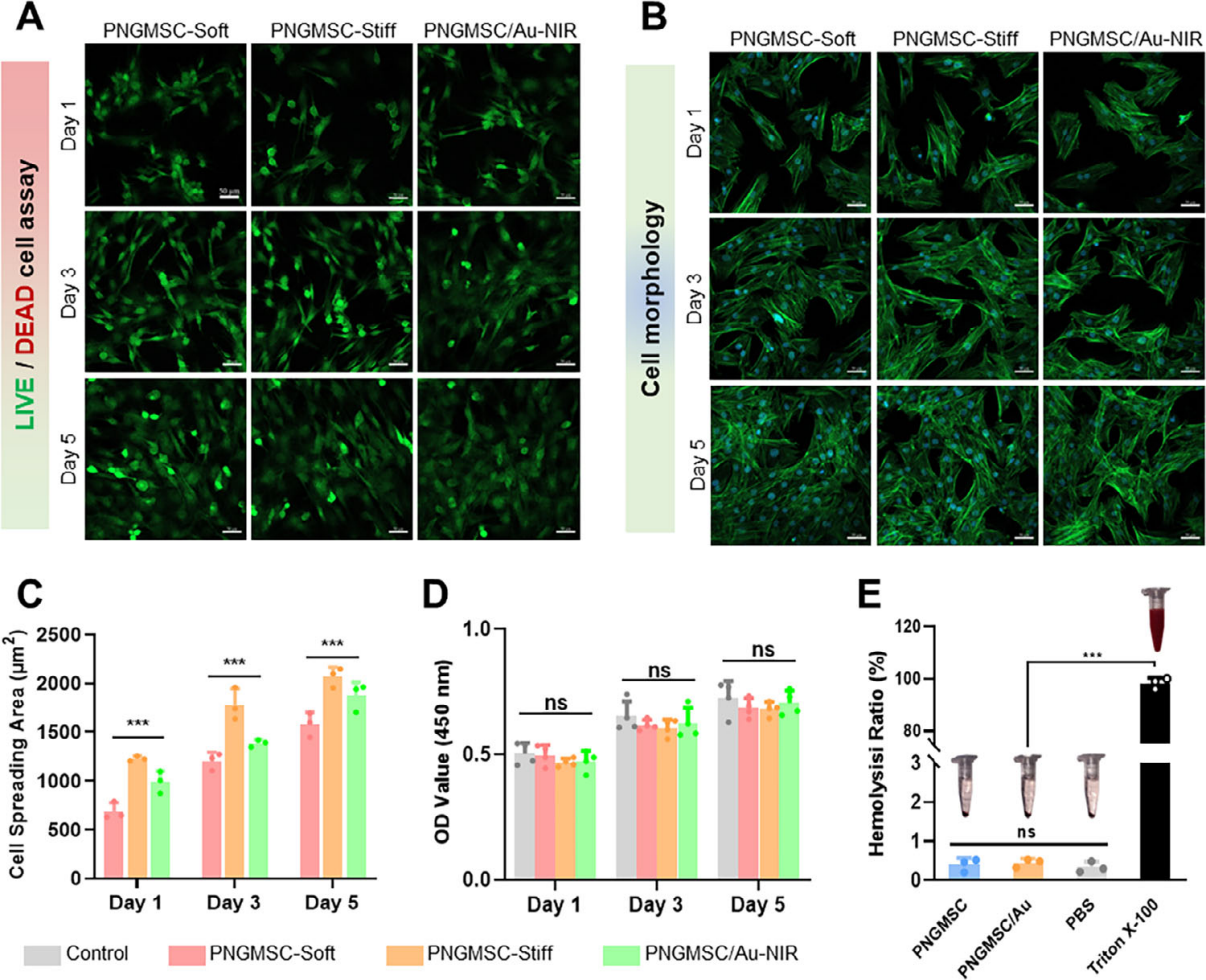
Biocompatibility evaluation of the dynamic stiffness hydrogel system. (A) Live/dead staining images of BMSCs cultured on different hydrogels for 1, 3, and 5 days, showing high cell viability across all samples (scale bar = 50 µm, n = 3). (B) Cytoskeletal and nuclear staining (phalloidin/DAPI) demonstrating stiffness‐dependent changes in BMSC morphology at days 1, 3, and 5 (scale bar = 50 µm, n = 3). (C) Quantification of cell spreading area on hydrogels with different stiffnesses at days 1, 3, and 5 (n = 3). (D) CCK‐8 assay showing proliferation of BMSCs cultured on hydrogels over 5 days (n = 3). (E) Hemolysis assay evaluating the blood compatibility of PNGMSC and PNGMSC/Au hydrogels, demonstrating hemolysis ratios <1% (n = 3). Data are presented as mean ± SD. Statistical significance: ns, *p* > 0.05; ^*^ *p* < 0.05; ^**^ *p* < 0.01; ^***^ *p* < 0.001.

### 2.4 Influence of Hydrogel Stiffness on Progenitor Maintenance and Stem‐Cell–Associated Behaviors

The PNGMSC/Au‐NIR group was initially cultured under a soft mechanical condition (∼7 kPa). NIR irradiation was applied at Day 3 to trigger Ca^2^^+^ release and induce a stiffness increase to ∼23 kPa. During the early phase of bone repair, maintaining a pool of proliferative, multipotent progenitor cells is essential to support subsequent osteogenic commitment. Excessively rapid lineage specification may prematurely deplete this reservoir and compromise regenerative outcomes. Because BM‐MSCs are highly sensitive to mechanical cues in their microenvironment, substrate stiffness provides an effective means of regulating the balance between proliferation, progenitor maintenance, and lineage commitment.

Immunofluorescence staining revealed that Nanog and Oct4 expression was lowest in the PNGMSC‐Stiff group but was significantly increased in the PNGMSC/Au‐NIR group (Figure 4A), confirming that a dynamically stiffening microenvironment is more conducive to maintaining stem cell pluripotency and self‐renewal capacity. Consistently, β‐galactosidase staining showed a greater proportion of senescent BM‐MSCs in the PNGMSC‐Stiff group (Figure 4B), suggesting that increased matrix stiffness may accelerate cellular maturation or stress‐related senescence. The expression levels of transcription factors associated with progenitor maintenance (*Rex‐1*, *Nanog*, *Sox2*, and *Oct4*) were markedly reduced on PNGMSC‐Stiff hydrogels (Figure 4C). While these markers are not definitive indicators of stemness in BM‐MSCs, their downregulation aligns with the observed decrease in immunofluorescence staining of Nanog and Oct4 on stiffer matrices. Moreover, upregulation of cell‐cycle regulatory genes (*CCNA1*, *CCNA2*, *CCNB1*, *CCND1*, *CCNE1*, *CDK1*) in the stiff group (Figure 4D–G) indicates an accelerated cell‐cycle progression, consistent with increased cytoskeletal tension and enhanced mechanotransductive signaling under higher stiffness conditions.

**FIGURE 4:**
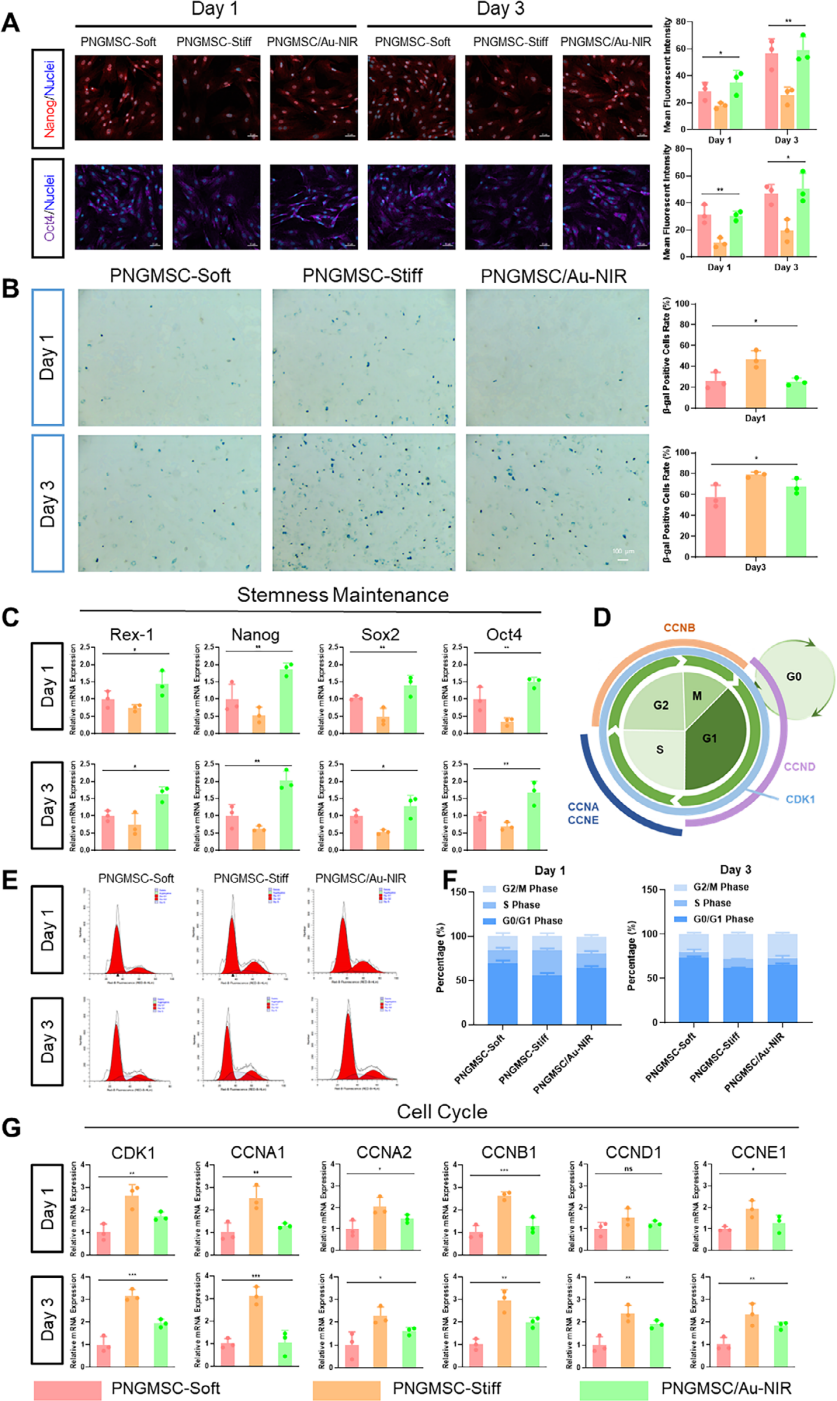
Regulation of stemness maintenance in BMSCs by the dynamic stiffness hydrogel system. (A) Immunofluorescence staining of Nanog and Oct4 in BMSCs cultured on hydrogels explanted from rat cranial defects on days 1 and 3, showing stiffness‐dependent stemness maintenance (scale bar = 50 µm, n = 3). (B) Senescence‐associated β‐galactosidase staining and quantification of β‐gal‐positive BMSCs after culture on hydrogels for 1 and 3 days (scale bar = 100 µm, n = 3). (C) qPCR analysis of pluripotency‐related genes (*Rex‐1*, *Nanog*, *Sox2*, *Oct4*) in BMSCs cultured on hydrogels at days 1 and 3 (n = 3). (D) Schematic of the cell cycle regulatory pathways involved in stiffness‐mediated stemness modulation. (E,F) Flow cytometry analysis and quantitative comparison of cell cycle phase distribution in BMSCs cultured on hydrogels with different stiffnesses (n = 3). (G) Expression levels of cell cycle–related genes (*CDK1*, *CCNA1*, *CCNA2*, *CCNB1*, *CCND1*, *CCNE1*) assessed via qPCR (n = 3). For the PNGMSC/Au‐NIR group, NIR irradiation was performed on Day 3, and measurements were conducted after stiffness transition. Data are presented as mean ± SD. Statistical significance: ns, *p* > 0.05; ^*^ *p* < 0.05; ^**^ *p* < 0.01; ^***^ *p* < 0.001.

As shown in Figure S2, BM‐MSCs in softer microenvironments also retained higher multipotent differentiation potential, whereas stiffer matrices favored early lineage commitment. This behavior can be attributed to inhibited cytoskeletal reorganization and reduced stress fiber formation under low stiffness, resulting in decreased intracellular tension. Such conditions restrict YAP/TAZ nuclear localization and maintain BM‐MSCs in a more progenitor‐like state. In contrast, increased stiffness enhances actin bundling and focal adhesion maturation, thereby activating mechanotransductive pathways that drive commitment toward osteogenic phenotypes. Alternatively, increased matrix stiffness enhances focal adhesion formation and cytoskeletal tension, which are subsequently transmitted to the nucleus to regulate mechanosensitive signaling pathways such as YAP/TAZ, thereby linking macroscopic mechanics to cell fate regulation [44]. These findings demonstrate that matrix stiffness exerts a strong regulatory influence on BM‐MSC pluripotency, self‐renewal capacity, senescence, and differentiationassociated signaling. The distinct cellular responses elicited by soft versus stiff matrices provide critical guidance for designing dynamically stiffening hydrogels capable of supporting early progenitor maintenance while promoting osteogenesis during later stages of bone repair.

### 2.5 In Vitro Regulation of Osteogenic Behavior by Dynamic Hydrogel Stiffness

All subsequent biological analyses were performed after irradiation, and the final stiffness level was comparable to that of the static PNGMSC‐Stiff group, while differing only in the temporal evolution of mechanical cues. During the early phase of bone regeneration, BM‐MSCs must preserve sufficient proliferative capacity and multipotent potential to meet the subsequent demands for osteogenic commitment. Once exposed to an appropriate mechanical microenvironment, these cells undergo lineage specification and initiate mineralized matrix production. Matrix stiffness is a well‐established regulator of osteogenic fate, with intermediate‐to‐high stiffness promoting osteoblast differentiation through mechanotransductive pathways [21, 22, 23].

In this study, the dynamically stiffening PNGMSC/Au‐NIR hydrogel provided an in situ transition from a compliant to a stiffer microenvironment, enabling staged regulation of BM‐MSC behavior. qPCR analysis demonstrated significantly elevated expression of osteogenesis‐associated genes—including *RUNX2*, *ALP*, *OCN*, and *OPN—*in the PNGMSC/Au‐NIR group (Figure 5D). These markers collectively represent sequential stages of osteogenic differentiation: *RUNX2* as an early transcriptional regulator, *ALP* as a marker of early matrix maturation, and *OCN/OPN* as indicators of late mineralization and matrix remodeling. Functional assays corroborated these findings. Alizarin Red S staining (Figure 5B) revealed minimal mineral deposition on PNGMSC‐Soft hydrogels, whereas PNGMSC/Au‐NIR exhibited the most robust calcium accumulation, consistent with the mechanically stiffened niche. ALP staining (Figure 5A) followed a similar trend. Immunofluorescence analysis further showed markedly enhanced RUNX2 and OPN expression in the PNGMSC/Au‐NIR group (Figure 5C), validating the superior osteogenic induction capacity of the dynamically stiffened microenvironment.

**FIGURE 5:**
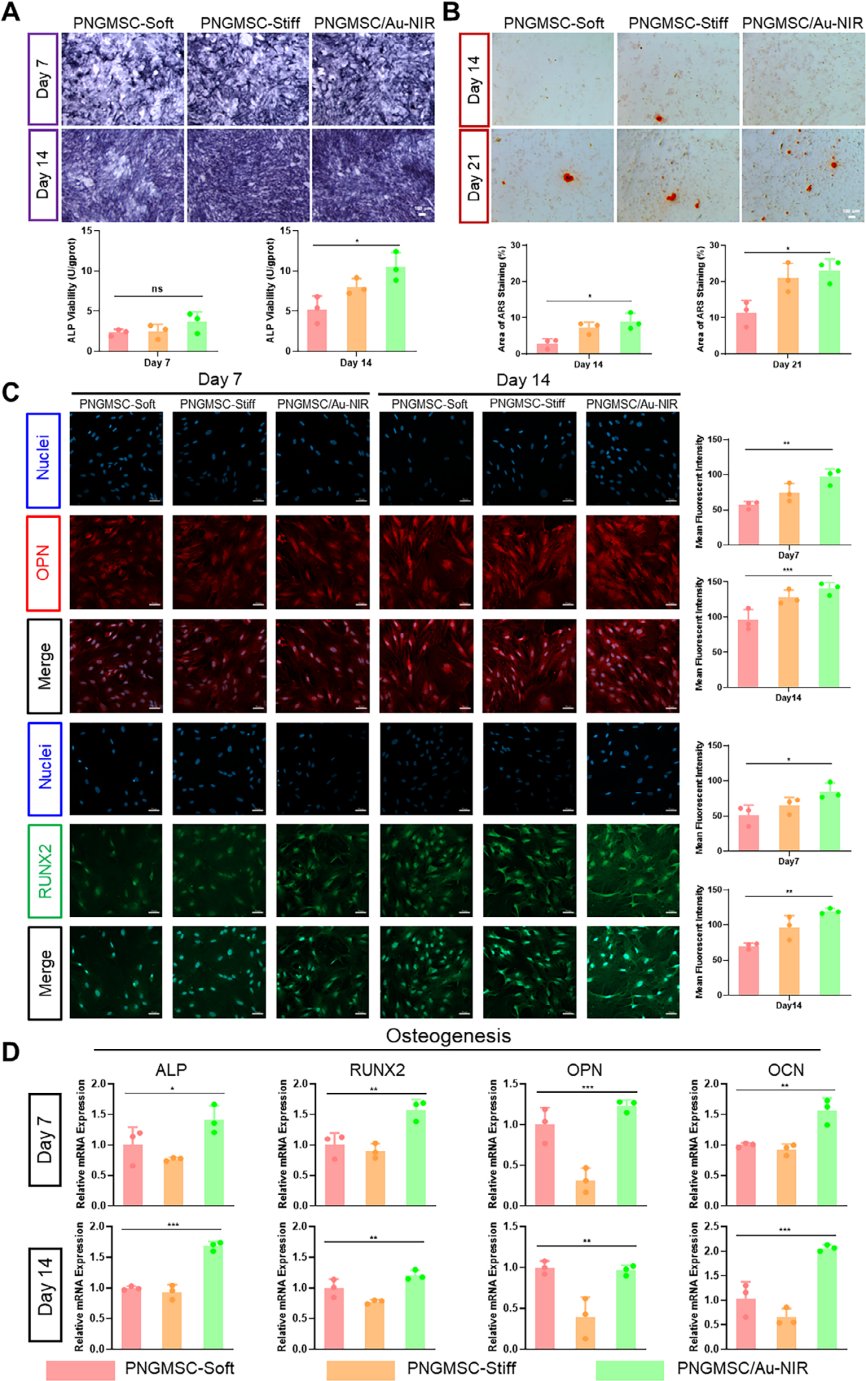
Dynamic stiffness–mediated osteogenic differentiation of BMSCs in vitro. (A) ALP staining of BMSCs after 7 and 14 days of osteogenic induction on hydrogels with different stiffnesses, with corresponding quantitative ALP activity analysis (scale bar = 100 µm, n = 3). (B) Alizarin Red S staining of calcium deposition at days 14 and 21, along with semi‐quantitative ARS extraction analysis (scale bar = 100 µm, n = 3). (C) Immunofluorescence staining of RUNX2 and OPN in BMSCs cultured on hydrogels of varying stiffness, with quantitative fluorescence intensity measurements (scale bar = 50 µm, n = 3). (D) qPCR assessment of osteogenesis‐related gene expression (*ALP*, *RUNX2*, *OPN*, *OCN*) at days 7 and 14, demonstrating enhanced osteogenic commitment under dynamically stiffened conditions (n = 3). Data are presented as mean ± SD. Statistical significance: ns, *p* > 0.05; ^*^ *p* < 0.05; ^**^ *p* < 0.01; ^***^ *p* < 0.001.

To exclude potential confounding effects from free Ca^2^^+^ ions present in the hydrogel, osteogenic differentiation was evaluated using hydrogel extracts (Figure S3). No significant influence on osteogenic markers was observed, confirming that the differentiation outcomes were primarily governed by matrix stiffness rather than ion leakage. Mechanistically, increased stiffness promotes cytoskeletal tension and organized stress‐fiber formation, which serve as critical upstream regulators of osteogenic commitment. Higher matrix stiffness facilitated the development of well‐defined actin stress fibers, enhancing cellular sensitivity to mechanical stimuli. This effect is consistent with the activation of mechanoresponsive pathways. Specifically, stiffer substrates promote nuclear translocation of YAP/TAZ, which subsequently upregulates RUNX2 expression and simultaneously activates ERK1/2 signaling, which enhances ALP and OPN expression. Together, these pathways converge to accelerate osteogenic maturation and matrix mineralization [45]. Overall, these results demonstrate that dynamic, NIR‐induced stiffening of the PNGMSC hydrogel provides a controllable mechanical microenvironment that effectively regulates BM‐MSC osteogenic behavior in vitro. The staged transition from soft to stiff resembles the natural progression of ECM stiffening during bone healing and offers a biomimetic strategy for modulating progenitor cell fate.

### 2.6 In Vivo Stiffness Regulation Promotes Bone Regeneration

Critical‐sized cranial defects were created in SD rats to evaluate whether NIR‐mediated in situ stiffening of the PNGMSC hydrogel could enhance bone regeneration (Figure 6A). Prior to assessing therapeutic efficacy, biosafety was confirmed through H&E staining of major organs at week 8, showing no detectable toxicity associated with the implanted hydrogels (Figure S4). A major consideration is whether Ca^2^^+^ release, and thus stiffness enhancement, could occur spontaneously at physiological temperature. In our system, Ca^2^^+^ is encapsulated within PNIPAm‐b‐PCL micelles and additionally stabilized by ionic coordination with SA. At 37°C, the micelles remain below their phase‐transition threshold and retain a compact core–shell structure, preventing Ca^2^^+^ leakage. Moreover, SA–Ca^2^^+^ complexes exhibit high coordination stability at physiological temperature, providing an additional thermodynamic barrier against ion dissociation. Only when the hydrogel undergoes localized NIR‐induced heating to 39–40°C does partial micelle disassembly occur, enabling Ca^2^^+^ release and subsequent reinforcement of the SA–Ca^2^^+^ coordination network. Thus, stiffness enhancement is dependent on NIR‐mediated photothermal activation and does not occur in untreated PNGMSC/Au hydrogels in vivo.

**FIGURE 6:**
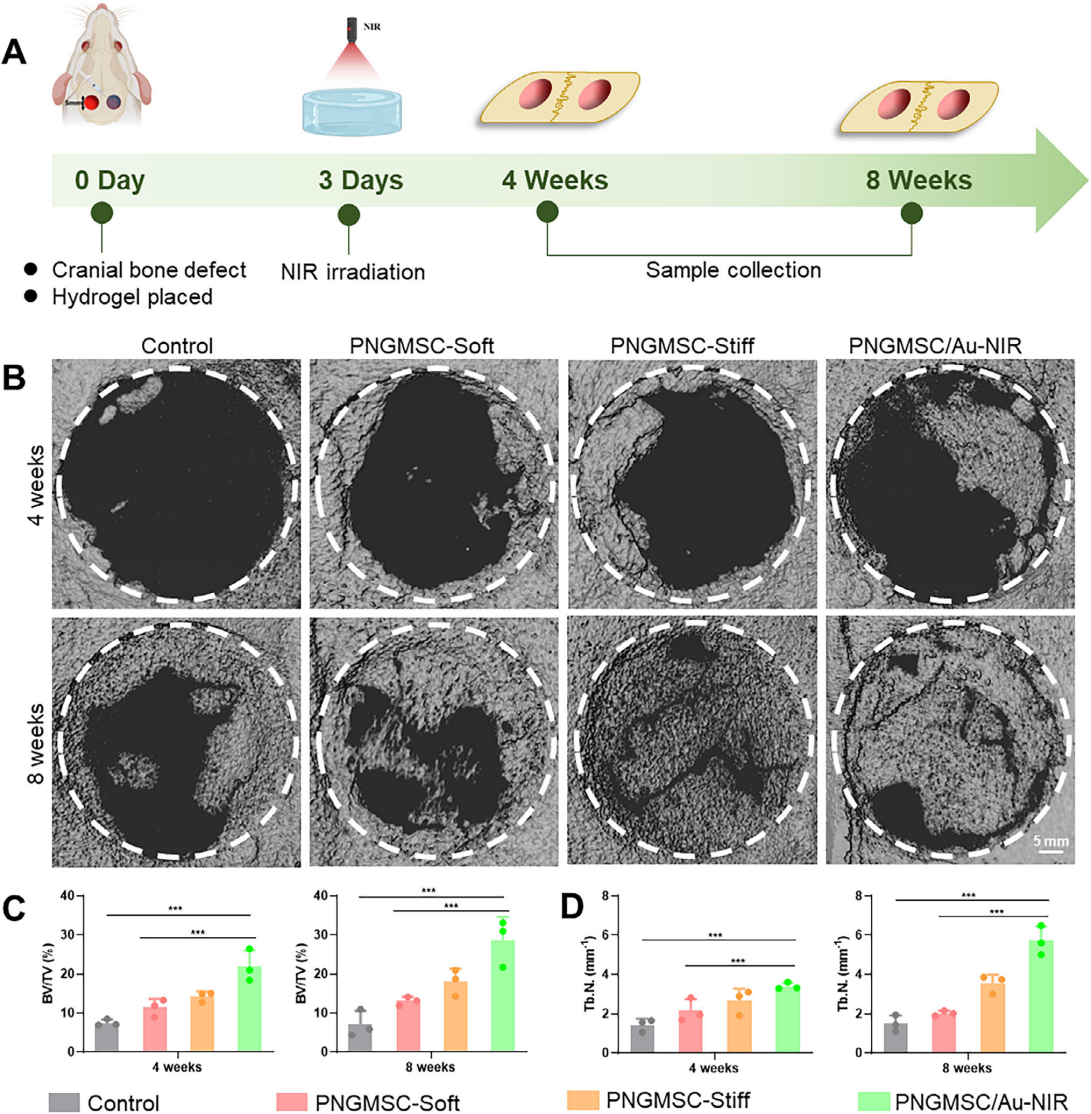
Micro‐CT evaluation of in vivo bone regeneration in critical‐sized cranial defects. (A) Schematic illustration of the in vivo experimental design and hydrogel implantation strategy. (B) Representative 3D micro‐CT reconstructions of rat cranial defects at 4 and 8 weeks following implantation of different hydrogel systems (scale bar = 5 mm, n = 3). (C,D) Quantitative analysis of new bone formation, including bone volume fraction (BV/TV) and trabecular number (Tb.N), demonstrating stiffness‐dependent enhancement in bone regeneration (n = 3). Data are presented as mean ± SD. Statistical significance: ns, *p* > 0.05; ^*^ *p* < 0.05; ^**^ *p* < 0.01; ^***^ *p* < 0.001.

Micro‐CT 3D reconstruction revealed progressive defect filling across all treated groups, with distinct differences in bone regeneration depending on hydrogel stiffness (Figure 6B). Quantitative analysis of BV/TV and trabecular number (Figure 6C,D) confirmed that stiffer hydrogels promoted more substantial bone formation. PNGMSC‐Soft facilitated early cell infiltration and bridging but supported limited mineralized tissue deposition. PNGMSC‐Stiff enhanced osteogenic differentiation but did not achieve complete bone integration. In contrast, PNGMSC/Au‐NIR—capable of transitioning from a soft to a stiff state in vivo—exhibited the highest degree of bone regeneration at both 4 and 8 weeks, reflecting the benefit of staged mechanical modulation. Micro‐CT scanning and 3D reconstruction can be used to visualize bone resorption [46, 47]. Quantitative micro‐CT analysis further confirmed that the PNGMSC/Au‐NIR group exhibited significantly higher BV/TV and improved trabecular architecture compared with static groups, providing robust radiographic evidence for the enhanced bone regeneration induced by dynamic stiffness regulation. H&E and Masson staining (Figure 7A,D) consistently demonstrated that all hydrogel structures remained well‐positioned within the defect region. In the blank group, defects remained largely filled with fibrous tissue even at week 8. PNGMSC‐Soft supported collagen deposition but resulted in incomplete bridging. PNGMSC‐Stiff allowed bone ingrowth but left regions of unmineralized fibrous tissue. Strikingly, PNGMSC/Au‐NIR defects were filled with continuous lamellar bone, and osteoblasts were aligned along the scaffold interface, forming cortical‐like structures indicative of advanced tissue maturation.

**FIGURE 7:**
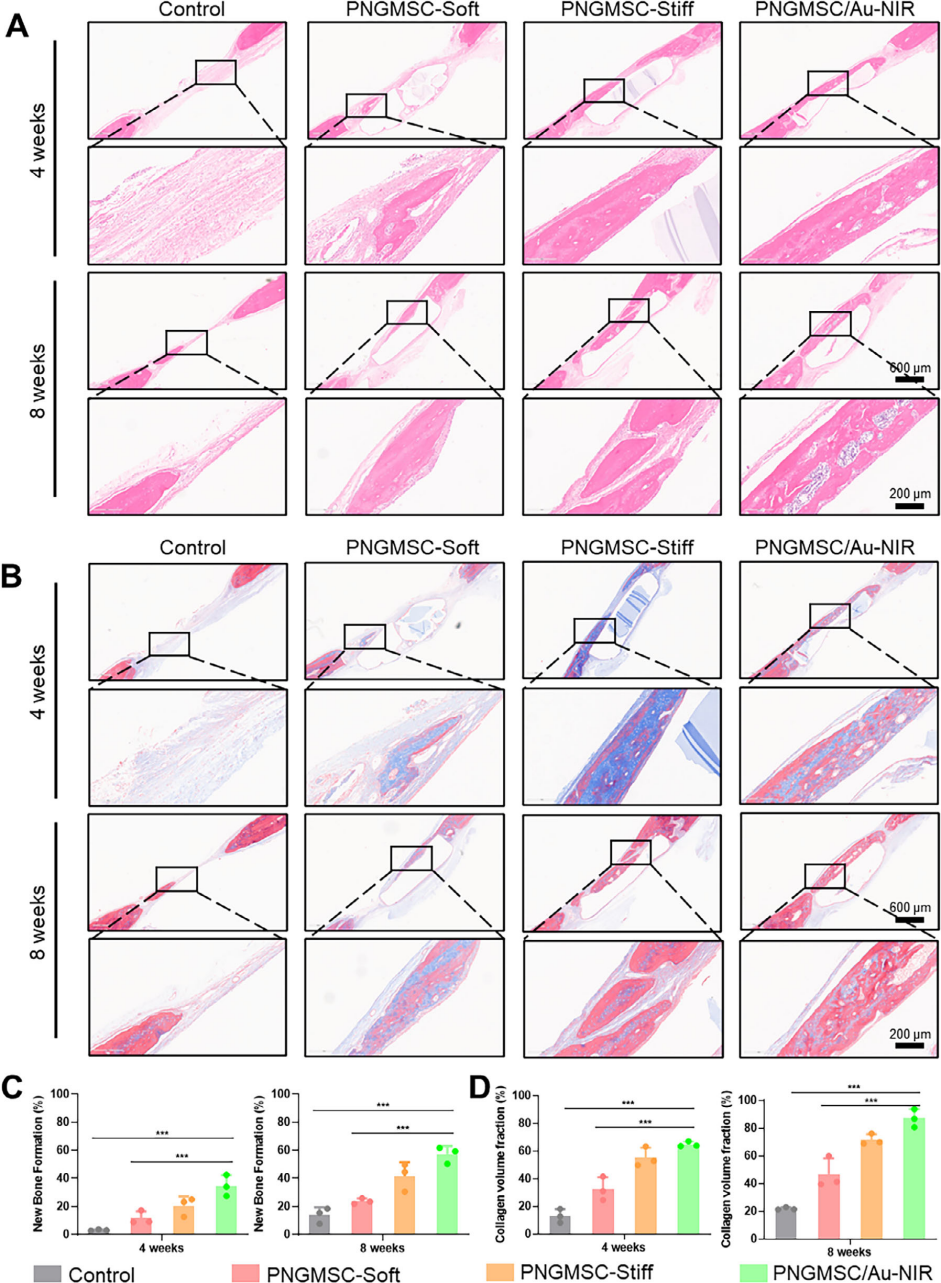
Histological assessment of bone regeneration and extracellular matrix deposition. (A) Representative H&E‐stained sections of rat cranial defects at 4 and 8 weeks post‐implantation; the nuclei appear blue‐purple, and cytoplasm/extracellular matrix appear pink. (scale bars = 600 µm and 200 µm). (B) Masson's trichrome staining illustrating collagen deposition and maturation in regenerated tissues at 4 and 8 weeks; collagen fibers and newly formed bone matrix are stained blue, while cytoplasm and muscle‐like tissues are stained red and nuclei appear dark. (scale bars = 600 µm and 200 µm). (C) Quantitative analysis of the percentage of newly formed bone within the defect region (n = 3). D) Quantification of collagen volume fraction in the defect area, reflecting ECM remodeling and matrix maturation (n = 3). Data are presented as mean ± SD. Statistical significance: ns, *p* > 0.05; ^*^ *p* < 0.05; ^**^ *p* < 0.01; ^***^ *p* < 0.001.

Immunohistochemical staining for RUNX2 and OPN (Figure 8A,D) further confirmed the superior osteogenic performance of the dynamically stiffened hydrogel. PNGMSC/Au‐NIR exhibited the highest proportion and intensity of RUNX2‐ and OPN‐positive cells at both 4 and 8 weeks. RUNX2 expression reflects early osteogenic commitment, while OPN marks active mineralization and matrix remodeling. Their strong and uniformly distributed expression in the PNGMSC/Au‐NIR group indicates sustained osteogenic activity and coordinated tissue maturation. Collectively, these results demonstrate that NIR‐triggered in situ stiffening is essential for achieving robust bone regeneration, as physiological temperature alone does not induce Ca^2^^+^ release or stiffness changes. By dynamically matching the evolving biomechanical requirements of the healing microenvironment‐ soft for early cell recruitment, stiff for osteogenic maturation‐ the PNGMSC/Au‐NIR hydrogel significantly enhances bone formation in critical‐sized defects. This strategy provides a programmable and clinically translatable approach for treating large bone defects.

**FIGURE 8:**
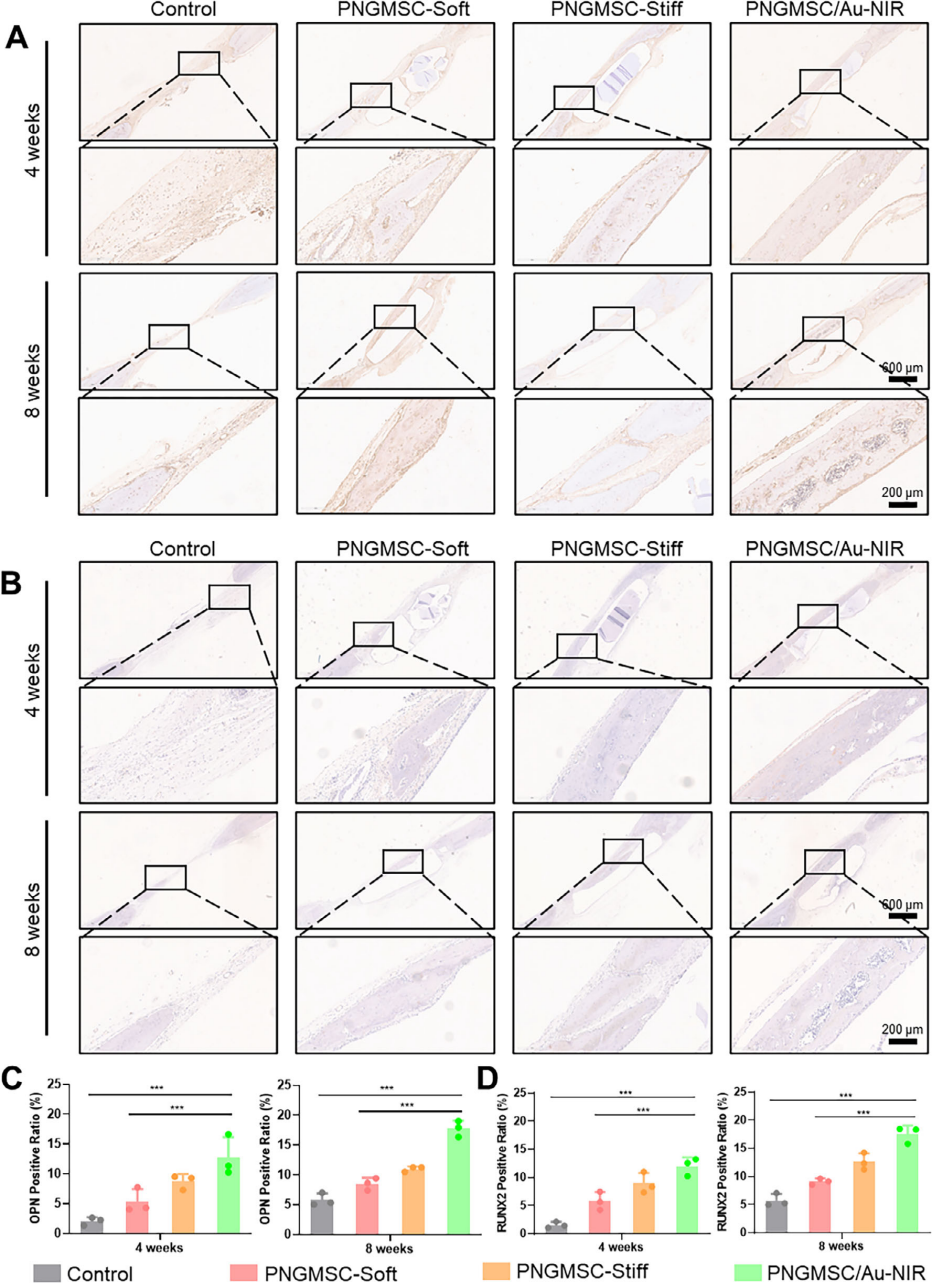
Immunohistochemical evaluation of osteogenic marker expression in vivo. (A) Representative images of OPN immunohistochemical staining in cranial defect sections at 4 and 8 weeks, demonstrating mineralization‐related matrix protein expression (scale bars = 600 µm and 200 µm). (B) Representative RUNX2 immunohistochemical staining at 4 and 8 weeks, indicating early‐stage osteogenic commitment (scale bars = 600 and 200 µm). The positive protein expression is indicated by brown coloration, while cell nuclei are counterstained blue with hematoxylin. (C,D) Quantitative analysis of OPN‐positive and RUNX2‐positive cells within the defect region at both time points (n = 3). Data are presented as mean ± SD. Statistical significance: ns, *p* > 0.05; ^*^ *p* < 0.05; ^**^ *p* < 0.01; ^***^ *p* < 0.001.

## 3 Conclusion

In this work, we developed a dynamically stiffening hydrogel that enables programmable mechanical modulation to recapitulate the evolving extracellular matrix environment during bone repair. By integrating BACA‐modified gold nanoparticles and calcium‐loaded thermoresponsive micelles within a dual interpenetrating polymer network, the system achieves NIR‐triggered, in situ stiffening that cannot occur under physiological temperature alone. This photothermally controlled release of Ca^2^^+^ allows precise enhancement of the SA–Ca^2^^+^ coordination network, providing phase‐appropriate biomechanical cues throughout the healing process. Comprehensive in vitro studies demonstrated that early low stiffness supports stemness maintenance and prevents premature exhaustion of the progenitor pool, whereas later‐stage stiffness promotes cytoskeletal maturation, osteogenic signaling activation, and robust mineralization. In vivo, the dynamically stiffened hydrogels significantly enhanced bone formation, accelerated defect bridging, and facilitated the development of mature lamellar bone in critical‐sized defects—outperforming both static soft and static stiff scaffolds. Collectively, this study presents a programmable and biomimetic materials platform capable of synchronizing scaffold mechanics with the natural timeline of bone regeneration. The ability to modulate stiffness on demand offers a powerful strategy to optimize stem cell fate, coordinate tissue maturation, and improve therapeutic outcomes. This dynamic stiffness hydrogel system holds strong potential for clinical translation in the treatment of challenging bone defects and may serve as a generalizable paradigm for mechanobiology‐informed regenerative biomaterials.

## Author Contributions

**Fan Yang**: methodology, validation, software, formal analysis, resources, conceptualization. **Yuxin Yang**: writing – original draft, investigation, methodology, software, data curation. **Lu Wang**: investigation, visualization, formal analysis, supervision, data curation. **Zongtai Li**: data curation, formal analysis, investigation, methodology. **Weichang Li**: writing – review and editing, writing – original draft, funding acquisition, supervision, resources, project administration, conceptualization. **Xinchun Zhang**: conceptualization, writing – review and editing, writing – original draft, funding acquisition, validation, software, supervision.

## Conflicts of Interest

The authors declare no conflict of interest.

## Supporting information




**Supporting File**: advs76920‐sup‐0001‐SuppMat1.docx.

## Data Availability

The data that support the findings of this study are available in the supplementary material of this article.
