# Supplementary material for: In Situ Programmable Modulation of Hydrogel Stiffness for Stage‐Adaptive Bone Regeneration
Source: Adv Sci (Weinh). 2026 Jul 31;13(60):e76920. doi: 10.1002/advs.76920 (PMC13427231; doi:10.1002/advs.76920)
Supplement: Supplementary file 1 [file ADVS-13-e76920-s001.docx]

Supporting Information

**In Situ Programmable Modulation of Hydrogel Stiffness for Stage-Adaptive Bone Regeneration**

Yuxin Yang, Fan Yang, Lu Wang, Zongtai Li, Weichang Li*, Xinchun Zhang*

Experimental Section

*Materials*: Acrylamide (AAm, 99%), N-isopropylacrylamide (NIPAm, 98%), anhydrous calcium sulfate (CaSO_4_, 97%), SA, gelatin, methacrylic anhydride (94%), sodium bicarbonate (99.9%), N,N'-methylenebis(acrylamide) (MBAA), ammonium persulfate (APS, 98.5%), N,N'-bis(acryloyl)cystamine (BACA, 98%), acetic acid-Sodium(＞80%, pH = 4.5), calcium chloride anhydrous (CaCl_2_, 99.9%), photoinitiator 2959 (98%), polycaprolactone were purchased from Macklin (Shanghai, China). Sulfo SANPAH, south American fetal bovine serum (FBS), DMEM/ F-12 culture medium, penicillin-streptomycin solution (P/S), phosphate-buffered saline (PBS, pH = 7.4), DAPI were purchased from Thermo-Fisher Scientific (USA). 1 M HEPES (pH = 7.2~7.4, sterile), alizarin red S (ARS) solution (1%, pH = 4.2), 1% Triton X-100 were purchased from Solarbio (Beijing, China). Calcium colorimetric assay kit, senescence β-galactosidase staining kit, actin-tracker green, BCIP/NBT alkaline phosphatase color development kit were purchased from Beyotime (Shanghai, China). Crystal violet (0.1%) was purchased from Aladdin (USA). β-glycerophosphate disodium salt hydrate, dexamethasone, vitamin C, bovine serum albumin (BSA) were purchased from Sigma-Aldrich (USA). Paraformaldehyde (3.7 wt%) was purchased from BioSharp Biotechnology (Anhui, China). Collagen I rat tail was purchased from

Corning (USA). Calcein-AM/propidiumiodide double staining kit was purchased from Bestbio (Shanghai, China). Alkaline phosphatase assay kit was purchased from Jiancheng (Jiangsu, China). RUNX2 antibody and osteopontin antibody were purchased from Affinity Biosciences (Australia). Nanog antibody and Oct4 antibody were purchased from Abcam plc (UK). Nano gold colloid was purchased from XFNANO (Jiangsu, China). Cetylpyridinium chloride was purchased from Adamas-beta (Shanghai, China). Prime-script RT reagent Kit and SYBR Premix EX Taq were purchased from TaKaRa Biotechnology (Japan). Cell Counting Kit-8 was purchased from DOJINDO laboratories (Japan).

**Synthetic method of hydrogel systems**

*Synthesis of GelMA:* Gelatin (10% w/v) was dissolved in PBS at 55 °C under continuous stirring until completely solubilized. Methacrylic anhydride (5 mL) was then added dropwise, and the reaction proceeded for 120 min at 55 °C. The mixture was diluted with warm PBS (40 °C) and subsequently dialyzed (MWCO 12–14 kDa) against deionized water for 7 days to remove unreacted methacrylic anhydride and low-molecular-weight impurities. The dialysate was centrifuged to remove aggregates, frozen, and lyophilized to obtain GelMA.

*Preparation of Au@BACA Nanoparticles:* N,N′-bis(acryloyl)cystamine (BACA) was added to an aqueous dispersion of gold nanoparticles (AuNPs) and sonicated continuously to facilitate the formation of BACA-modified AuNPs (Au@BACA). The modification relies on Au–S coordination and surface adsorption of acrylamide groups, enabling subsequent covalent crosslinking within the hydrogel.

*Preparation of Calcium-Loaded PNIPAm-b-PCL Micelles:* PNIPAm-b-PCL was dissolved in tetrahydrofuran (THF), followed by solvent removal under vacuum to obtain a thin polymer film. An aqueous CaSO₄ solution was added, and the mixture was stirred to induce micellization and encapsulation of Ca²⁺ ions. Excess calcium was removed through dialysis (MWCO 3.5 kDa) until no detectable Ca²⁺ remained in the external solution.

*Preparation of PNGMSC Hydrogels:* AAm, NIPAm, and GelMA were dissolved in deionized water to form Solution A. Sodium alginate (SA) was added to obtain the prepolymer mixture. Solution B was prepared by dissolving ammonium persulfate (APS), photoinitiator Irgacure 2959, and CaSO₄ in water. Solutions A and B were mixed thoroughly and exposed to UV irradiation to initiate free-radical polymerization, resulting in formation of the interpenetrating PNIPAm/GelMA/SA-Ca²⁺ hydrogel (PNGMSC). To generate hydrogels with different stiffnesses, Ca²⁺ concentrations were adjusted to 0.1%, 0.3%, 0.6%, or 1% (w/v), thereby modulating the density of SA–Ca²⁺ ionic crosslinks.

*Preparation of PNGMSC/Au and Dynamic Stiffening Hydrogels:* For hydrogels capable of in situ stiffness modulation, Ca²⁺-loaded PNIPAm-b-PCL micelles and Au@BACA were incorporated into Solution B before polymerization. PNGMSC/Au hydrogels were prepared using the same UV-polymerization procedure. Upon NIR irradiation, Au@BACA generated localized photothermal heating, inducing the thermal transition of micelles and triggering Ca²⁺ release. The released ions increased the SA–Ca²⁺ coordination crosslinking density, enabling in situ modulation of matrix stiffness.

**Physicochemical Characterization**

The morphology of calcium-loaded thermoresponsive micelles was examined using transmission electron microscopy (TEM, Tecnai G2 Spirit, 120 kV, FEI, Netherlands) before and after NIR stimulation. To quantify calcium release kinetics, micelle suspensions were mixed with an equal volume of 5% Triton X-100 to lyse the micelles (control group), while experimental groups were exposed to NIR irradiation (808 nm, 2 W cm⁻²). Calcium concentrations were measured using a calcium colorimetric assay kit according to the manufacturer’s protocol. The morphology and size distribution of Au nanoparticles and Au@BACA were characterized using TEM, and the presence of sulfur-containing functional groups on AuNP surfaces was confirmed by energy-dispersive X-ray spectroscopy (EDS). The micromechanical properties of hydrogels were evaluated using atomic force microscopy (AFM, Bruker, USA). Polystyrene microspheres (radius: 2.5 μm) were attached to MLCT-O10 V-shaped cantilevers, and force–distance curves were collected in fluid using NanoScope Analysis 2.0 software. Young’s modulus was calculated via Hertzian contact modeling. Hydrogels were cryofractured in liquid nitrogen and lyophilized to preserve their microstructure for field-emission scanning electron microscopy (SEM, Zeiss Sigma 300). Freeze-drying was intentionally employed to prevent vacuum-induced collapse of hydrated hydrogel networks. Cross-sectional pore morphology was analyzed using ImageJ. Surface roughness was quantified with a laser scanning confocal microscope (LSM 700, Zeiss, Germany), using the arithmetic mean height (Sa) and maximum height (Sz) parameters. Water contact angles were measured using a DSA-XROLL goniometer to assess hydrogel surface hydrophilicity. For swelling behavior, lyophilized hydrogels were immersed in PBS (pH 7.4) at 37 °C, and weight changes were recorded at predetermined intervals to evaluate water uptake kinetics.

**Biocompatibility**

*Collagen Coating of Hydrogels:* Hydrogels were cut into discs using a sterile biopsy punch and rinsed three times with 1 M HEPES. After removing excess buffer, a UV-activatable crosslinker solution (0.2 mg/mL sulfo-SANPAH in PBS) was added, and samples were exposed to 365 nm UV light for 30 min. Hydrogels were washed thoroughly, followed by incubation with type I collagen (rat tail, 50–100 µg/mL) at 37 °C for 2 h. After incubation, collagen was removed and discs were rinsed three times with sterile PBS. Collagen coating was intentionally applied to equalize cell-adhesion ligand density across hydrogels with different stiffness, thereby ensuring that differences in cell behavior were attributable to matrix mechanics rather than surface chemistry variations.

*Isolation and Culture of BMSCs:* Bone marrow–derived mesenchymal stromal cells (BMSCs) were harvested from 7–13-day-old male Sprague–Dawley rats. Following cervical dislocation and alcohol sterilization, femurs were excised and flushed with DMEM/F-12 supplemented with 2% penicillin/streptomycin (P/S). The collected cell suspension was centrifuged (1500 rpm, 5 min), and the pellet was resuspended in DMEM/F-12 with 10% FBS and 1% P/S. Cells were seeded onto collagen-coated hydrogels and cultured at 37 °C with 5% CO₂. Third-passage (P3) BMSCs were used in all experiments.

*Live/Dead Staining:* Cell viability (days 1, 3, 5) was assessed using a Calcein-AM/PI Live/Dead kit. After washing with PBS, hydrogels were incubated with the staining solution for 30 min in the dark and imaged using a laser scanning confocal microscope (LSM980, Zeiss).

*Cell Proliferation:* BMSCs were seeded at 1×10⁵ cells/well in 48-well plates containing hydrogel samples. On days 1, 3, and 5, CCK-8 reagent was added and incubated for 2 h. Absorbance at 450 nm was recorded using a microplate reader (Thermo 3001, USA).

*Cell Morphology:* At designated time points, cells on hydrogels were fixed, permeabilized, and stained with Alexa Fluor 488-phalloidin and DAPI. Cytoskeletal morphology was imaged by confocal microscopy (LSM980, Zeiss).

*Hemolysis Assay:* Rat blood was collected using heparin anticoagulant and centrifuged at 4 °C to isolate RBCs. A 20-fold diluted RBC suspension was mixed with PNGMSC, PNGMSC/Au, PBS (negative control), and 0.1% Triton X-100 (positive control). After 1 h incubation at 37 °C, samples were centrifuged and absorbance of the supernatant was measured at 540 nm to calculate hemolysis rates.

**Effects of Hydrogel Substrate on Stem Cell Stemness**

*Immunofluorescence staining of stemness markers:* At days 1 and 3, cells were fixed and permeabilized with 0.1% Triton X-100. After blocking with 10% BSA for 1 h, the samples were incubated with primary antibodies against Nanog and Oct4 at 4 °C for 24 h. Following washing with PBS, the cells were incubated with Alexa Fluor 555‑conjugated secondary antibodies and counterstained with DAPI. Images were acquired using a laser‑scanning confocal microscope (LSM980, Zeiss), and fluorescence intensity was quantified with ImageJ software.

*β-Galactosidase (SA-β-Gal) Staining:* Cells cultured on hydrogels were washed with PBS, fixed with 3.7% paraformaldehyde, and incubated with SA-β-gal staining solution (X-Gal substrate). Senescent cells (blue) were imaged and quantified using a fluorescence stereomicroscope (MZ10F, Leica).

*Flow Cytometry for Cell Cycle Analysis:* Cells were fixed in 70% ethanol and stored overnight at 4 °C. After resuspension in PI staining buffer, samples were incubated at 37 °C for 30 min and analyzed using a Guava® easyCyte™ flow cytometer. Cell cycle distribution was determined using ModFit LT software.

*RNA Extraction and qRT-PCR:* Total RNA was isolated using the RNA-Quick Purification Kit and reverse-transcribed using the PrimeScript™ RT Reagent Kit. Gene expression of stemness-associated cell-cycle regulators (*CDK1*, *CCNA1*, *CCNA2*, *CCNB1*, *CCND1*, etc.) was quantified using an ABI QuantStudio 5 real-time PCR system. Relative expression levels were calculated using the 2⁻ΔΔCt method. Primer sequences are listed in Table S1.

**Effect of Hydrogel Dynamic Stiffness Modulation on Osteogenic Behavior In Vitro**

*Osteogenic Induction:* To evaluate the osteogenic differentiation of BMSCs on hydrogels with different stiffness states, cells were cultured in osteogenic induction medium consisting of DMEM/F-12 supplemented with 10% FBS, 1% P/S, 10 mM β-glycerophosphate, 0.1 μM dexamethasone, and 50 μM ascorbic acid. Medium was refreshed every two days.

*Alizarin Red S (ARS) Staining and Quantification:* After 14 and 21 days of induction, cells were rinsed with PBS and fixed with 4% paraformaldehyde for 20 min. Samples were incubated with ARS staining solution for 30 min at room temperature in the dark. Excess dye was removed with distilled water, and calcium deposition was imaged using a stereofluorescence microscope (MZ10F, Leica, Germany). For quantitative analysis, cetylpyridinium chloride (CPC) was used to dissolve bound ARS. The resulting solution was centrifuged, and absorbance of the supernatant was measured at 562 nm using a microplate reader (Biotek Synergy H1, USA).

*Alkaline Phosphatase (ALP) Staining and Activity Assay:* On days 7 and 14, cells were washed, fixed, and stained using a BCIP/NBT ALP staining kit following the manufacturer’s protocol. Stained samples were imaged using a stereofluorescence microscope (MZ10F, Leica). ALP enzymatic activity was further quantified using an ALP assay kit, and absorbance values were measured using a microplate reader.

*Immunofluorescence Staining of Osteogenic Markers:* At days 7 and 14 of induction, cells were fixed and permeabilized with 0.1% Triton X-100. After blocking with 10% BSA for 1 h, samples were incubated with primary antibodies against RUNX2 and OPN at 4 °C for 24 h. Following PBS washes, cells were incubated with Alexa Fluor 555-conjugated secondary antibodies and counterstained with DAPI. Images were acquired using a laser scanning confocal microscope (LSM980, Zeiss), and fluorescence intensity was quantified using ImageJ software.

*qRT-PCR Analysis of Osteogenic Gene Expression:* Total RNA was extracted as described in the previous section. cDNA was synthesized using a reverse transcription kit, and qRT-PCR was performed to analyze the expression of osteogenic markers (*RUNX2*, *ALP*, *OCN*, *OPN*) using an ABI QuantStudio 5 system. Gene expression levels were calculated using the 2⁻ΔΔCt method. Primer sequences are listed in Table S1.

**In vivo Stiffness Modulation for Enhanced Bone Regeneration**

*Rat Bone Defect Model:* A total of 48 male Sprague–Dawley rats (200 ± 20 g) were provided by the Laboratory Animal Center of Sun Yat-sen University. All procedures were approved by the Institutional Animal Care and Use Committee (SYSU-IACUC-2023003499). Rats were randomly assigned to four groups (n = 6 per group). Anesthesia was induced using 3% sodium pentobarbital (40 mg/kg, Sigma, USA) combined with 10% chloral hydrate (300–400 mg/kg, Sigma, USA). A sagittal midline incision was made to expose the calvarium, and a standardized 5-mm critical-sized cranial defect was created using a low-speed handheld drill under saline irrigation. Each rat received two defects, allowing implantation of two different hydrogel samples in one animal. This ensured that for each hydrogel condition, at least three independent biological replicates (n = 3) were collected per time point by conducting experiments in parallel across a sufficient number of animals. Following implantation, incisions were sutured, and penicillin was administered for three consecutive days. Postoperative vital signs were monitored closely. Notably, PNGMSC/Au hydrogels remained at physiological temperature (37 °C) in vivo and did not release Ca²⁺ spontaneously. Due to the phase-transition behavior of PNIPAm-b-PCL micelles, Ca²⁺ release and subsequent stiffening occur only when the local temperature rises above ~38–39 °C. Therefore, targeted NIR irradiation was applied to trigger photothermal heating of Au nanoparticles, enabling controlled in situ stiffness modulation.

*Micro-CT Evaluation:* At 4 and 8 weeks post-implantation, rat calvaria were harvested and scanned using a high-resolution micro-CT system (μCT 50, SCANCO Medical AG, Switzerland) at 70 kVp, 114 μA, and 10 μm voxel size. 3D reconstruction, bone volume fraction (BV/TV), and trabecular thickness (Tb.Th) were analyzed using Amira-Avizo software to quantify in vivo new bone formation.

*Histological and Immunohistochemical Analysis:* Harvested samples were rinsed in PBS, fixed in 4% paraformaldehyde, and decalcified in 10% EDTA before paraffin embedding and sectioning. H&E staining was used to evaluate tissue morphology and scaffold–tissue integration. Masson’s trichrome staining assessed collagen deposition and new bone matrix formation. Immunohistochemistry (IHC) for osteocalcin (OCN) and other bone markers was performed to evaluate osteogenic activity and maturation. Images were acquired using a Leica Aperio AT2 digital slide scanner. Quantification of positive staining and percentage of marker-positive cells was performed using ImageJ software.

*Statistical Analysis:* All quantitative results were obtained from at least three independent biological samples (n ≥ 3) and reported as mean ± standard deviation (SD). Differences between two groups were analyzed using an unpaired two-tailed Student’s t-test. For multiple comparisons, data normality was evaluated using the Shapiro–Wilk test, followed by one-way ANOVA with Bonferroni post hoc correction when appropriate. Statistical analyses were conducted using GraphPad Prism 8.0. Significance levels were defined as: ns, *p* > 0.05; **p* < 0.05; ***p* < 0.01; ****p* < 0.001.

**Table S1.** The primer sequences used in this study.

| **Gene** | **Direction** | **Sequences** |
| --- | --- | --- |
| ***Rex-1*** | Forward | 5’ AAGACCTTTCTTGCCAGGTT 3’ |
|  | Reverse | 5’ CCACCCTTTAGCTTCTCTCC 3’ |
| ***Nanog*** | Forward | 5’ GACTAGCAACGGCCTGACTCA 3’ |
|  | Reverse | 5’ CTGCAATGGATGCTGGGATA 3’ |
| ***Sox2*** | Forward | 5’ ATTACCCGCAGCAAAATGAC 3’ |
|  | Reverse | 5’ TTTTTGCGTTAATTTGGATGG 3’ |
| ***Oct4*** | Forward | 5’ CCCCATTTCACCACACTCTACTC 3’ |
|  | Reverse | 5’ GTGACAGGAACAGAGGGAAAGG 3’ |
| ***GAPDH*** | Forward | 5’ GACATGCCGCCTGGAGAAAC 3’ |
|  | Reverse | 5’ AGCCCAGGATGCCCTTTAGT 3’ |
| ***CDK1*** | Forward | 5’ CAGGACTCCAGGCTGTATCTCAT 3’ |
|  | Reverse | 5’ CTTATCGGTATTCCAAACGCTCT 3’ |
| ***CCNA1*** | Forward | 5’ GCCCGATGTCGATGAGTTTGTCT 3’ |
|  | Reverse | 5’ GAAGGAGGAACTGGTTGGTGGTT 3’ |
| ***CCNA2*** | Forward | 5’ GTATTTGCCATCGCTTATTGCTG 3’ |
|  | Reverse | 5’ TGCTGTGGTGCTTTGAGGTAGGT 3’ |
| ***CCNB1*** | Forward | 5’ GAACGGCTGTTAGTGTTTAGGTG 3’ |
|  | Reverse | 5’ TCTTGACTGTTCGCTGACTTTATT 3’ |
| ***CCND1*** | Forward | 5’ GAGCAGAAGTGCGAAGAGGAGGT 3’ |
|  | Reverse | 5’ GGCGGATAGAGTTGTCAGTGTAGAT 3’ |
| ***CCNE1*** | Forward | 5’ GTGCTACTTGACCCACTGGACTC 3’ |
|  | Reverse | 5’ ACGACCACTCGCCGTACCCTATC 3’ |
| ***β-actin*** | Forward | 5’ GGCTCCTAGCACCATGAAGAT 3’ |
|  | Reverse | 5’ AAGGGTGTAAAACGCAGCTC 3’ |
| ***RUNX2*** | Forward | 5’ TCCGCCACCACTCACTACCAC 3’ |
|  | Reverse | 5’ GGAACTGATAGGACGCTGACGAAG 3’ |
| ***ALP*** | Forward | 5’ GCCTACTTGTGTGGCGTGAA 3’ |
|  | Reverse | 5’ AGGATGGACGTGACCTCGTT 3’ |
| ***OCN*** | Forward | 5’ GCCCTGACTGCATTCTGCCTCT 3’ |
|  | Reverse | 5’ TCACCACCTTACTGCCCTCCTG 3’ |
| ***OPN*** | Forward | 5’ CCAAGCGTGGAAACACACAGCC 3’ |
|  | Reverse | 5’ GGCTTTGGAACTCGCCTGACTG 3’ |
| ***GAPDH*** | Forward | 5’ CCGCATCTTCTTGTGCAGTG 3’ |
|  | Reverse | 5’ ATCCGTTCACACCGACCTTC 3’ |

Figure S1. In vivo photothermal effect of PNGMSC/Au hydrogel. A) Thermal imaging of hydrogel at the rat cranial defect site under NIR irradiation. B) Statistical analysis of surface temperature changes on the rat skull (n=3). C) The PNGMSC/Au hydrogel was subjected to an additional Ca²⁺ release experiment at 37°C for 72 h, followed by statistical analysis of the release percentage (n = 3).

**Figure S2**. In vitro adipogenic differentiation performance. Oil Red O staining of PNGMSC-Soft, PNGMSC-Stiff, and PNGMSC/Au-NIR samples (bar = 200 μm).

**Figure S3**. Osteogenic differentiation effects of hydrogel extracts. A) ARS of BMSCs cultured in A hydrogel extract after 7 days of osteogenic induction (bar = 100 μm). B, C) ALP staining and quantitative analysis of ALP activity in BMSCs cultured in A hydrogel extract after 7 days of osteogenic induction (bar = 100 μm, n = 3). D) qPCR analysis of relative mRNA expression levels of osteogenic genes (*ALP*, *RUNX2*, *OPN*, *OCN*) on Day 7 (n = 3). ns, *p* > 0.05, **p* < 0.05, ***p* < 0.01, ****p* < 0.001.

**Figure S4.** In vivo biocompatibility assessment. Representative H&E staining images of the main organs of the rat cranial defect at 8 weeks post-operation (bar= 200 μm).
